# Supplementary figures and images for: Incidence and predictors of preterm neonatal mortality at Mbarara Regional Referral Hospital in South Western Uganda
Source: PLoS One. 2021 Nov 2;16(11):e0259310. doi: 10.1371/journal.pone.0259310 (PMC8562818; doi:10.1371/journal.pone.0259310)

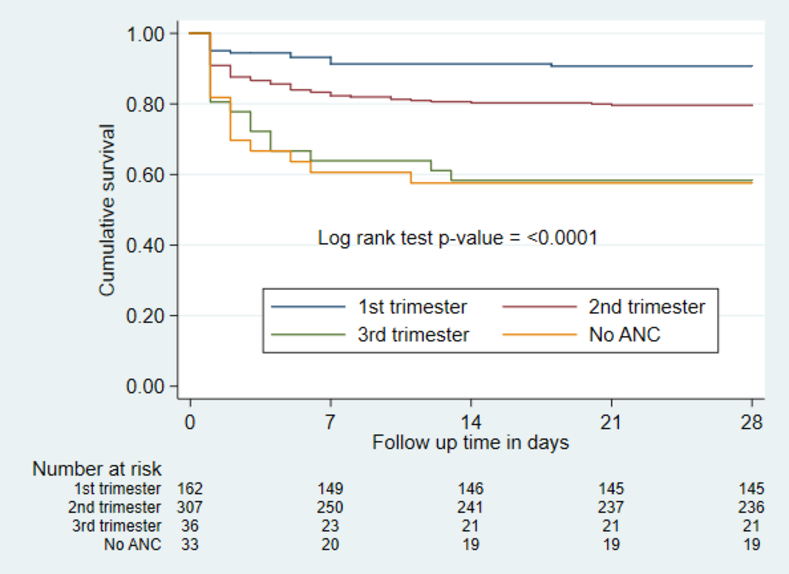

Supplement: S1 Fig — (TIF) [file pone.0259310.s001.tif]

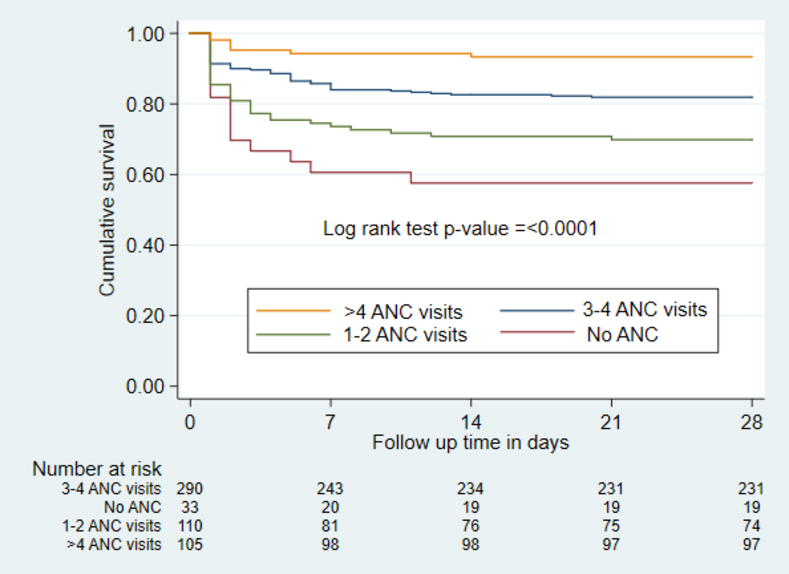

Supplement: S2 Fig — (TIF) [file pone.0259310.s002.tif]

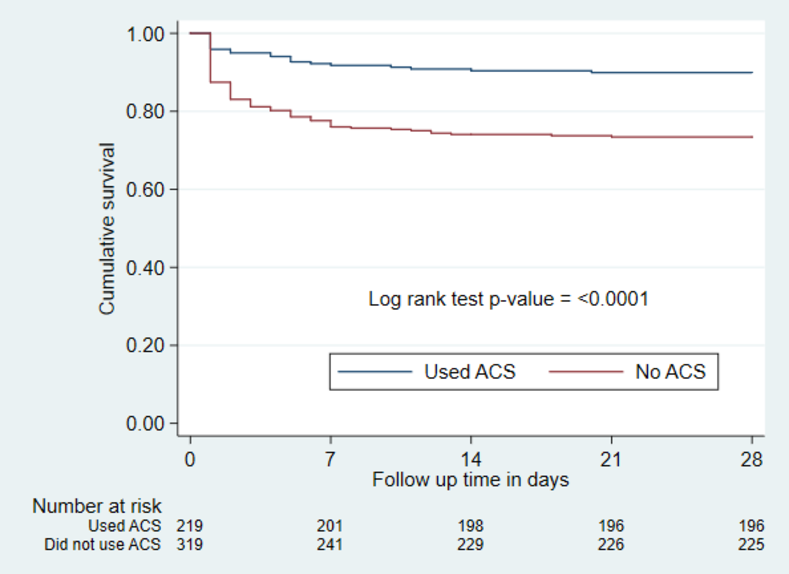

Supplement: S3 Fig — (TIF) [file pone.0259310.s003.tif]

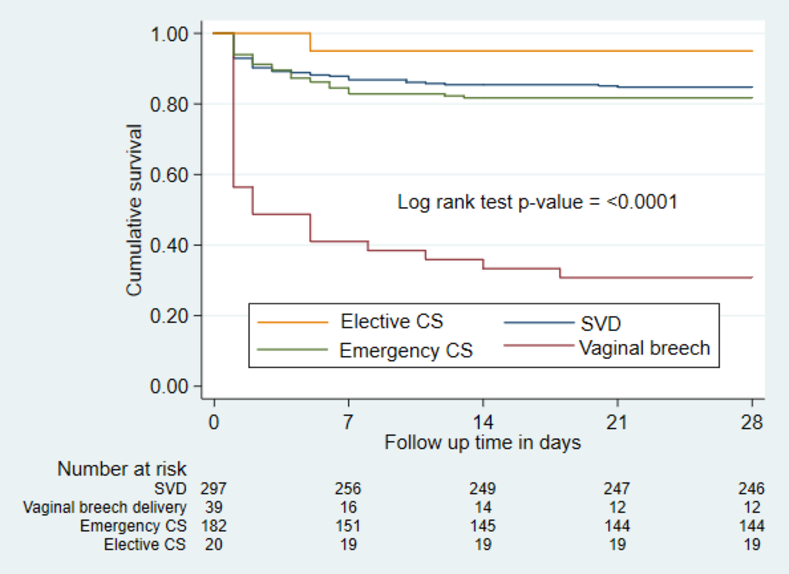

Supplement: S4 Fig — (TIF) [file pone.0259310.s004.tif]

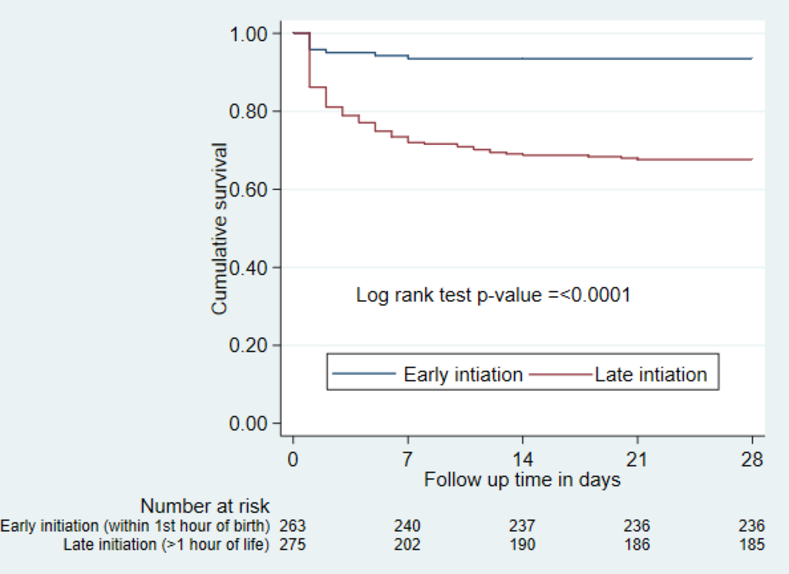

Supplement: S5 Fig — (TIF) [file pone.0259310.s005.tif]

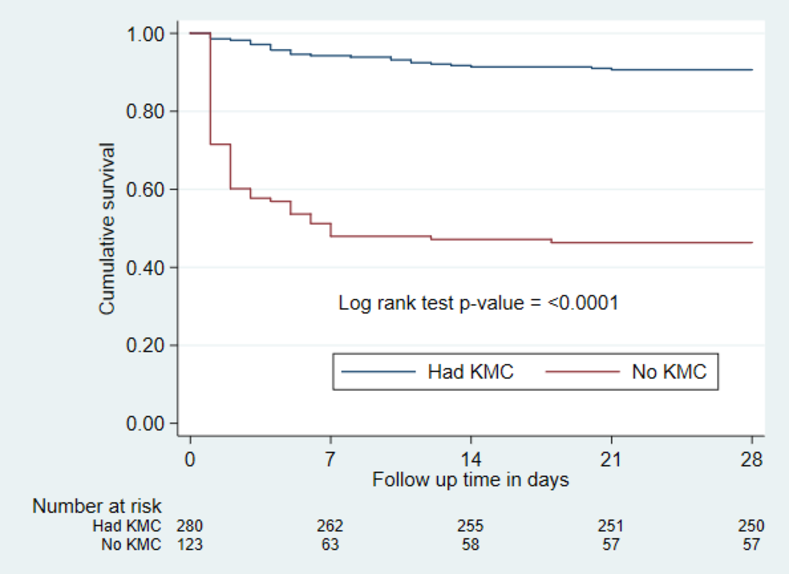

Supplement: S6 Fig — (TIF) [file pone.0259310.s006.tif]

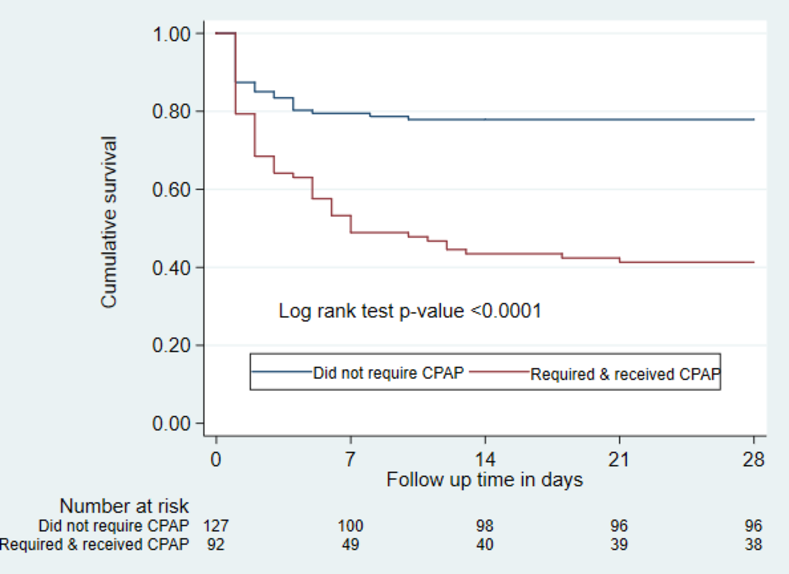

Supplement: S7 Fig — (TIF) [file pone.0259310.s007.tif]
