# Supplementary material for: Incidence and predictors of preterm neonatal mortality at Mbarara Regional Referral Hospital in South Western Uganda
Source: PLoS One. 2021 Nov 2;16(11):e0259310. doi: 10.1371/journal.pone.0259310 (PMC8562818; doi:10.1371/journal.pone.0259310)
Supplement: Attachment — Submitted filename: PLOS ONE COMMENTS FOR MANUSCRIPT.docx [file pone.0259310.s009.docx]

PLOS One

1. This is an important study on incidence of preterm neonatal mortality and predictors of mortality in a tertiary hospital in South Western Uganda. It highlighted the major predictors such as low antenatal attendance, Hypothermia and RDS and lack of Kangaroo mother care. The predicators identified could help to design appropriate interventions to reduce the preterm related Mortality in the Tertiary Insusitution.
2. The Abstract is well summarised , I would suggest improve the language in paragraph 1
3. The statistics	is Rigorous and appropriate for the Study design
4. **METHODS SECTION**
5. Did we exclude the extreme low birth weight , were all the babies at 28 weeks one kilo and above , is that true, what of the babies born to PET mothers , Did we exclude the extreme low birth weight, if so why, this may affect the mortality rates.

It is surprising all the babies were all above one kilo

6. Did we do Bilirubin levels done, or did we try to use a Kramer Chart – Please clearly state the method of measurement of the Bilirubin Levels
7. The babies that had Respiratory distress sydromme, was it all in the first week
8. What was their time frame ( Between first week ) or was this involving those after one week , If after one week how would you differentiate between other diseases like sepsis or apnea of Prematurity from Respiratory Distress syndrome, because RDS seems to account for many Deaths and yet diseases like sepsis , donot account for very few deaths, which is surprising
9. How would you diagnose Nosocomial pneumonia, were blood Cultures done , What do you mean , Could that be late one set sepsis
10. When was the Hypothermia Diagnosed at the Time of admission or during the course of admission

**Discussion**

11. Introduction could be improved , by highlighting the purpose of the study, instead of reporting all the important significant Results in the First Paragraph
12. The Respiratory Distress sydromme , assessment, we would have used the SAS score , it has been used in Kiwoko Hospital
13. When did the Hypothermia Occur, was it birth or during the admission, because hypothermia on admission to the NICu is associated with mortality. This could be added to the explanation for hypothermia
14. The difference in the mortality could be explained by the Different levels of care in neonatology ( refer to article Survive and thrive, Inpatient care for small and sick newbor barriers By Hannah Blencowe 2015 )
15. The different interventions available in Mbarara , how do they differ with other the hospitals
16. RDS – major cause of mortality , more studies from Mulago could explain that ( Yaser et al 2012)
17. More explanation for Hypothermia, what prevents Hypothermia, do we have a warm chain, what interventions are available for Hypothermia apart from kangaroo mother care
18. Need to explain, why those that needed CPAP, Died , do we have a criteria for those that have failed CPAP, if so what do we do , do we refer , we need to explain in the discussion
