# Supplementary material for: Incidence and predictors of preterm neonatal mortality at Mbarara Regional Referral Hospital in South Western Uganda
Source: PLoS One. 2021 Nov 2;16(11):e0259310. doi: 10.1371/journal.pone.0259310 (PMC8562818; doi:10.1371/journal.pone.0259310)
Supplement: Attachment — Submitted filename: RESPONSE TO REVIEWERS.docx [file pone.0259310.s011.docx]

**RESPONSE TO REVIEWERS’ COMMENTS**

**Reviewer #2: Most of the comments have been sufficiently addressed except the following**

1. **Labelling of the tables reviewer responded that "The tables are labelled as follows; table 1: sample size calculation (this is within the text), table 2: Baseline socio-demographic and obstetric characteristics, table 3: Characteristics of preterm neonates, table 4: Cumulative mortality incidence of preterm neonates, table 5: Predictors of mortality among preterm neonates born at Mbarara Regional Referral Hospital. However in the document table section they are labelled as table 2, table 1, table 2 then table 5, please correct.**

Response: This is noted and has been corrected. The tables are now labelled as follows; table 1: Sample size calculation, table 2: Baseline socio-demographic and obstetric characteristics, table 3: Characteristics of preterm neonates, table 4: Predictors of mortality among preterm neonates born at Mbarara Regional Referral Hospital.

2. **Wide confidence interval as this could results in loss of precision should at least be mentioned to caution the readers.**

Response: We acknowledge this limitation and have stated it as such. (Page/Line: 21/338-340)

3. **Based on the objective of the study "to determine the incidence and predictors of preterm neonatal mortality", I am not convinced that KM curves for each risk factor is necessary especially since they are not adjusted. Multivariate cox regression answer the question well. KM curves could be attached as supplementary material if needed.**

Response: We acknowledge that multivariable cox regression models answer the objective of this study and that KM curves are not adjusted. We have therefore attached the KM curves as supplementary material as advised.

**Reviewer #3: Well articulated manuscript on an important public health subject especially in the settings where the study is carried out. The paper highlights important interventions required to improve preterm outcomes.
Recommend it for publication and dissemination.**

Response: We appreciate the reviewer for the positive comments.
